# An aqueous two-phase system formed in single-component solution of α-ketooctanoic acid†

**DOI:** 10.1039/d1ra06474f

**Published:** 2021-10-21

**Authors:** Huifang Xu, Xin Liang, Yaping Zhang, Meihua Gao, Na Du, Wanguo Hou

**Affiliations:** College of Pharmacy, Henan University of Chinese Medicine Zhengzhou 450046 P. R. China; Key Laboratory of Colloid and Interface Chemistry (Ministry of Education), Shandong University Jinan 250100 P. R. China

## Abstract

Aqueous two-phase systems (ATPSs), consisting of two immiscible water-rich phases, have received great attention. So far, all of ATPSs reported are formed by two water-soluble compounds in aqueous media. Herein, we report an ATPS formed in the single-component aqueous solution of α-ketooctanoic acid (KOCOOH), a weakly acidic surfactant, without any additives. Its formation originates from the coexistence of micelles and vesicles in the system, the former existing in the upper phase and the latter in the lower phase. The phase behavior and microstructures of KOCOOH in aqueous solution were determined. A concentration-driven stepwise aggregation was identified for the KOCOOH solution. With an increase in the KOCOOH concentration, vesicles, oil droplets, micelles, strip bilayers, and planar lamellar phase form successively; macroscopically, the system exhibits a homogeneous transparent single-phase, turbid dispersion, two phases, a bluish single-phase, and a colorless transparent single-phase in turn. The constantly changing ionization state of KOCOOH in aqueous solution plays an important role in the phase and aggregate structure transition. This work deepens the understanding of ATPSs, and the ATPS formed by KOCOOH may have potential applications such as in the separation and purification of biomolecules and the construction of hierarchical protocell models.

## Abstract 2

An aqueous two-phase system can form in the single-component solution of α-ketooctanoic acid without any additives.

## Graphical abstract

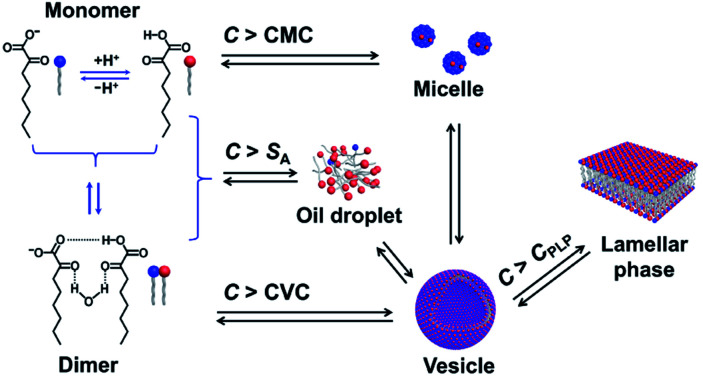

## 1. Introduction

Aqueous two-phase systems (ATPSs), consisting of two immiscible water-rich phases, have received great attention due to their fundamental and practical significance.^1–7^ The formation of ATPSs is closely related to the aggregation states of solutes in water,^1,8–14^ and research on their nature can thus deepen the understanding of aggregation phenomena in solution. The principle of ATPSs can be used to construct hierarchical protocells that may serve as a more realistic model of cellular organization.^15–17^ As one kind of potential separation media, ATPSs have been widely investigated in the separation of biomolecules,^1,3–5^ inorganic ions,^6,7^ and organic substances.^6,7,18^ In particular, owing to the high water content and low interfacial tension, ATPSs are conducive to maintaining the activity of biomolecules.^3^

A large variety of ATPSs have been discovered in aqueous solutions of two water-soluble compounds,^7^ such as two polymers, polymer/salt, alcohol/salt, oppositely charged surfactant/polyelectrolyte, two oppositely charged surfactants, ionic liquid (IL)/salt or polymer,^2,6^ and deep eutectic solvent (DES)/salt.^19–21^ The surfactant-based ATPSs are of great significance owing to their diversity in compositions and aggregate structures.^10^ However, there have been no reports on ATPSs formed in the single-component aqueous solution of surfactants without any additives.

Recently, we investigated the aggregation behavior of α-ketooctanoic acid (KOCOOH, also called 2-oxooctanoic acid), a derivative of the fatty acid (FA) octanoic acid, in water at 25 °C.^22^ It was demonstrated that KOCOOH itself can form vesicles with a low critical vesicle concentration (CVC, ∼1.4 mM) within a wide pH range (*ca.* 2–10). In appearance, KOCOOH solution was transparent at concentration (*C*) lower than 28 mM (the apparent solubility of KOCOOH, *S*_A_, at 25 °C), became turbid at *C* in 30–60 mM, and exhibited macroscopic phase separation at *C* > 60 mM. In recent experiments, we accidentally found that KOCOOH in water at *C* > 160 mM can form a homogeneous transparent (single-phase) system, which made us realize that an ATPS could form in the KOCOOH solution within an appropriate *C* range (*ca.* 60–160 mM). Notably, Xu *et al.*^11^ reported ATPSs of lauric acid (a fatty acid) in the presence of inorganic or organic alkalis (NaOH, CsOH, or (C_2_H_5_)_4_NOH); Arnould *et al.*^14^ also involved an ATPS formed in the mixture between the FA myristic acid and choline hydroxide. However, they both introduced specific cations to the FA solutions. In our case, only KOCOOH and water molecules participate in the construction of the ATPS with no extra cations introduced.

In the current work, the structures of aggregates formed in KOCOOH aqueous solutions with different concentrations were determined, to explore the formation mechanism of the ATPS. A concentration-driven stepwise aggregation was identified for the KOCOOH solution. The formation of the ATPS can be attributed to the coexistence of micelles and vesicles in the system, the former existing in the upper phase and the latter in the lower phase. To the best of our knowledge, this is the first time to find ATPSs formed in a single-component aqueous solution of surfactants without any additives.

## 2. Experimental section

### 2.1. Chemicals

α-Ketooctanoic acid (≥99% purity) was purchased from Sigma–Aldrich, China. All the reagents were of analytical reagent grade and used as received. Ultrapure water with a resistivity of 18.25 MΩ cm was obtained using a UPR-II-20T purification system (Sichuan ULUPURE Ultrapure Technology Co., China).

### 2.2. Sample preparation and phase state observation

Sample solutions were prepared by adding designed amounts of surfactants and water into 5 mL glass vials, and subsequently homogenized by vortex shaking. All the samples were transferred into an incubator and left to stand at 25.0 ± 0.1 °C for at least one month before measurements, to achieve sufficient aggregation equilibrium. To avoid possible photo-initiated reactions of alkyl ketoacids,^23^ the test samples were covered with aluminum foils and all measurements were conducted under indirect lighting.

The phase states of the test systems were determined by visual observation with the help of two crossed polarizers.

### 2.3. Measurements

#### 2.3.1. Dynamic light scattering (DLS)

DLS technique was used to determine the size distribution and mean hydrodynamic diameter (*D*_h_) of the aggregates. The measurements were performed at 25.0 ± 0.1 °C on a Zetasizer Nano ZS90 light scattering apparatus (Malvern, UK) equipped with a He–Ne laser (632.8 nm, 4 mW).

#### 2.3.2. Polarized optical microscopy (POM)

The POM micrographs of the lamellar phase of samples were taken by an OLYMPUS-BX53 POM with a CCD camera (OLYMPUS, Japan).

#### 2.3.3. Cryogenic transmission electron microscopy (cryo-TEM)

In a highly humid environment (>90%), an aliquot of 4 μL sample solution was dropped on a carbon-coated copper grid. Then, a thin liquid film was left on the grid by removing excess sample solution with two pieces of filter papers. About 10 s later, the resulted grid was quickly plunged into liquid ethane that was frozen by liquid nitrogen, and the vitrified sample was transferred into a specific cryogenic specimen holder and observed on a JEM-1400 TEM (JEOL, Japan).

#### 2.3.4. Small-angle X-ray scattering (SAXS)

The structural characterization of lamellar phase was performed by a SAXS instrument (SAXSess Anton-Paar, Austria) with a Kα radiation (0.154 nm) operating at 40 kV and 30 mA. The temperature was held constant using a standard temperature control unit (Anton-Paar TCS 120) connected with the SAXSess.

#### 2.3.5. pH measurements

The pH values of the solutions were determined at 25.0 ± 0.1 °C using a FE28 pH meter (Mettler Toledo, Sweden) with a LE422 glass micro-electrode. Three measurements were performed for each sample, and the average value was reported.

## 3. Results and discussion

### 3.1. Phase behavior

In our previous work,^22^ we reported the spontaneous formation of vesicles from KOCOOH aqueous solution with a CVC of *ca.* 1.4 mM. With increasing *C* to 28 mM, the KOCOOH system became turbid, and oil droplets were identified in the system. The present work focused on the phase behavior of KOCOOH solution with *C* above its *S*_A_ (∼28 mM).

**Fig. 1:**
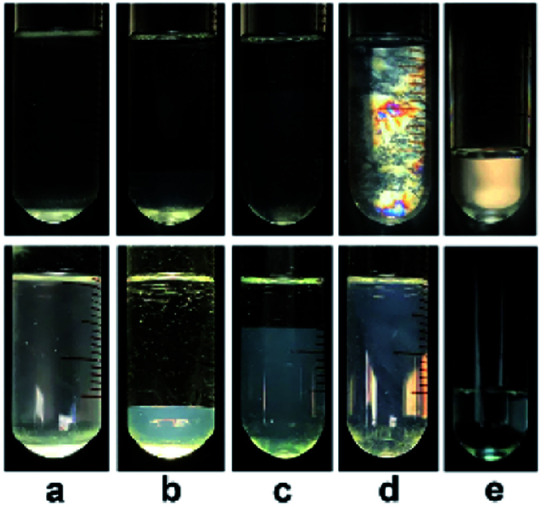
Photographs of KOCOOH solutions at (a) 60, (b) 100, (c) 130, (d) 250, and (e) 2000 mM, with (up) and without (below) crossed polarizers.

A *C*-dependent phase behavior was observed at 25.0 °C for KOCOOH solutions within a wide *C* range (28–2000 mM), by visual inspection with and without two crossed polarizers, as shown in Fig. 1 (and Fig. S1, ESI†). In the relatively low *C* range (28 ≤ *C* < 65 mM), the KOCOOH solutions were turbid and slightly bluish, and no obvious phase separation and birefringence were observed (Fig. 1a). As *C* increased to 65–160 mM, an ATPS with a clear interface was formed (Fig. 1b and c); the upper phase was colorless and transparent, and the lower phase was turbid and obviously bluish, but no obvious birefringence was observed for both the two phases. In addition, the volume of the lower phase increased with an increase in *C*. With a further increase in *C* to 160–2000 mM, the phase separation phenomena disappeared, and the system transformed into a homogeneous and birefringent single-phase (Fig. 1d and e). Note that the single-phase systems were bluish at 160 ≤ *C* ≤ 500 mM while became colorless at *C* > 500 mM.

So far, all of ATPSs reported are formed by two water-soluble compounds in aqueous media.^2,6,7,19–21^ In our case, the ATPS is formed in the single-component aqueous solution of KOCOOH without any additives. The formation of the ATPS indicates that special aggregation states exist in the KOCOOH solution.^1,8–14^

### 3.2. Structure of aggregates

The turbid systems with *ca.* 28–65 mM were identified in our previous work^22^ to be dispersions of oil droplets, indicating that microscopic phase-separation occurred although no macroscopic phase-separation was observed. Note that vesicles could be observed for the turbid systems (Fig. S2, ESI†), indicating that vesicles and oil droplets coexist in the systems. Our interest focused on the aggregate structures of the systems with higher *C*.

**Fig. 2:**
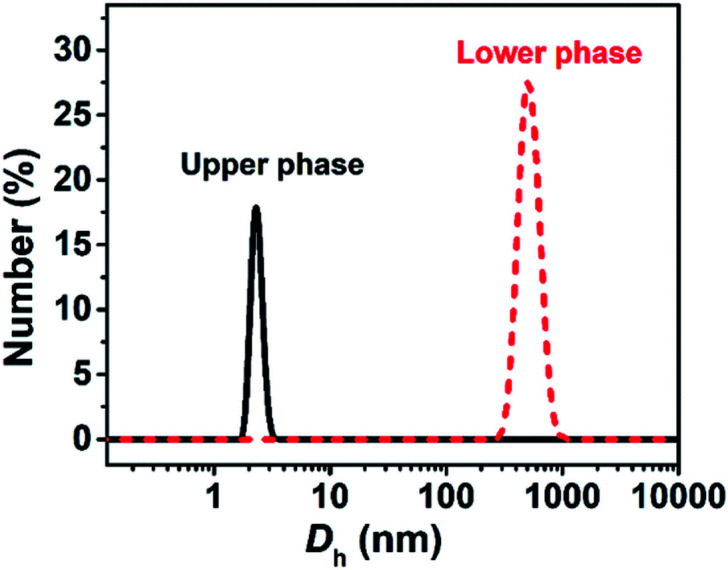
Size distributions of the upper and lower phases of the ATPS with 130 mM.

**Fig. 3:**
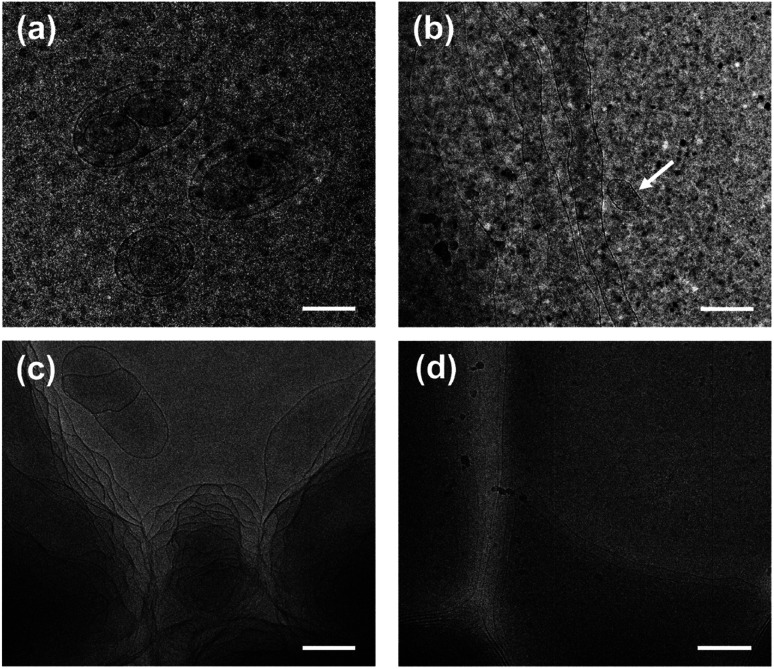
Cryo-TEM images of (a) the lower phase of ATPS at 130 mM and (b–d) the single-phase KOCOOH solutions at (b) 250, (c) 750 mM and (d) 2000 mM. Scale bar = 200 nm.

Each phase of the ATPS at 130 mM was detected by DLS (Fig. 2), indicating that aggregates exist in both the upper and lower phases, with *ca.* 2.2 and 500 nm in size, respectively. The small aggregates in the upper phase can be identified as micelles, which size is exactly twice the length of the fully extended KOCOOH molecule (∼1.09 nm (ref. 22)). The large aggregates in the lower phase may be vesicles, which were confirmed by cryo-TEM observations. Multi-compartment and multi-lamellar vesicles with *ca.* 300 nm in size were clearly observed for the lower phase (Fig. 3a), and only small spherical aggregates with ∼5 nm in size were observed for the upper phase (Fig. S3, ESI†). These results indicate that the critical micelle concentration (CMC) of KOCOOH in water is *ca.* 65 mM, and the micelles formed coexist with vesicles in the system. Owing to the density of KOCOOH being slightly higher than that of water, the system is separated into the upper and lower phases, consisting of micelles and vesicles, respectively. Therefore, it can be concluded that the formation of ATPSs observed here arises from the coexistence of micelles and vesicles in the system, similar to previous reports.^10,11,13,14^ Notably, for the ATPSs formed by FAs (lauric acid and myristic acid) and alkalis or choline hydroxide,^11,14^ the upper and lower phases were identified to be vesicle and micelle phases, respectively, contrary to our results. This is because the density of the FAs is slightly lower than that of water,^14^ contrary to the case of KOCOOH.

**Fig. 4:**
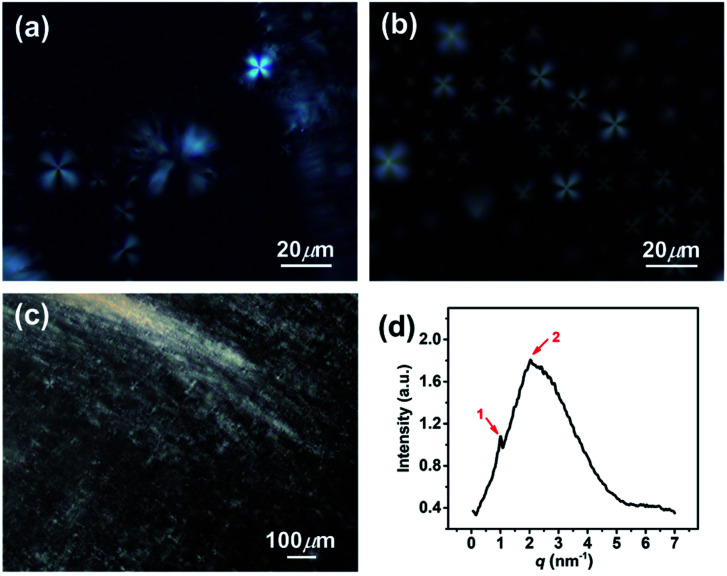
(a–c) Polarized optical micrographs and (d) SAXS pattern for the KOCOOH solutions with different concentration. (a) 750 mM, (b) 1000 mM, and (c and d) 2000 mM.

In addition, the homogeneous single-phase systems with 160–2000 mM were also detected using cryo-TEM. A large number of strip aggregates (bilayer sheets) along with a small number of vesicles (as indicated by arrows) were observed within 160–500 mM (Fig. 3b), and only a densely stacked planar lamellar structure was observed at *C* > 500 mM (Fig. 3c and d). The planar lamellar structure was further confirmed by POM and SAXS (Fig. 4). The Maltese crosses and Schlieren textures were observed for the systems at 750, 1000, and 2000 mM (Fig. 4a–c), evidenced the formation of planar lamellar phase.^10,11,24^ The SAXS pattern of the system at 2000 mM (Fig. 4d) shows two scattering peaks at *ca.* 1.02 (*q*_1_) and 2.04 (*q*_2_) nm^−1^, and the *q*_1_ : *q*_2_ ratio is 1 : 2, corresponding to the Bragg scattering of lamellar structures.^14^ The *d*-spacing of the lamellar structure was estimated from *d* = 2π/*q*_1_ to be *ca.* 6.16 nm. The larger *d* value than the thickness of the bilayers (∼2.1 nm (ref. 22)) indicates that a lot of water is trapped between the lamellar bilayers, similar to the previous reports.^14,25,26^

**Fig. 5:**
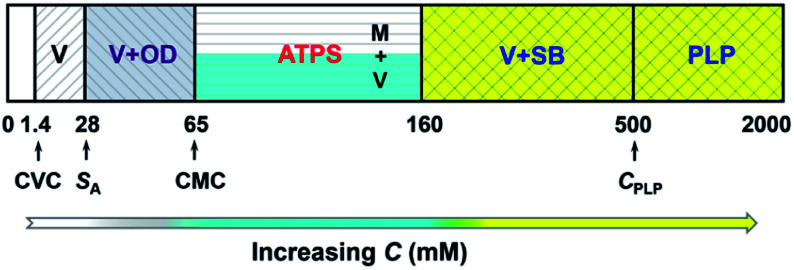
Concentration-dependent aggregation of KOCOOH in water at 25 °C. V: vesicles; OD: oil droplets; M: micelles; SB: strip bilayers; PLP: planar lamellar phase.

Based on the above results along with our previous work,^22^ it can be concluded that, microscopically, a concentration-driven stepwise aggregation occurs in the KOCOOH solution (Fig. 5). That is, an increase in *C* induces the formation of vesicles, oil droplets, micelles, strip bilayers, and planar lamellar phase in succession, in which the oil droplets, micelles, and strip bilayers formed all coexist with vesicles. Macroscopically, the system exhibit homogeneous transparent single-phase, turbid dispersion, two phases, bluish single-phase, and colorless transparent single-phase in turn.

### 3.3. Aggregation mechanism

**Fig. 6:**
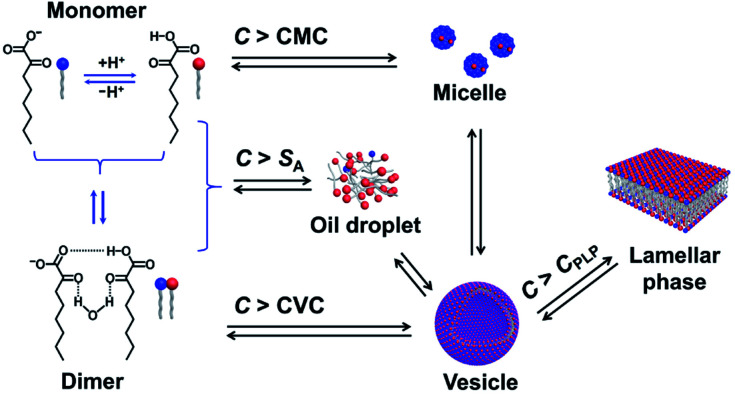
Schematic illustration for chemical equilibria and phase transition of KOCOOH in water. *C*_PLP_ represents the critical concentration at which the planar lamellar phase begins to form.

One question raised by the above results is why micelles are formed when the *C* exceeds its solubility. A possible reason is the existence of multiple species in the single-component system,^11,14,22^ which are in complex chemical equilibria, as shown in Fig. 6. KOCOOH is a weakly acidic surfactant, with a p*K*_a_ of ∼2.78.^22^ There exist three species in the system, *i.e.*, unionized (neutral acid, KOCOOH) and ionized (negatively charged soap, KOCOO^−^) species as well as the “acid-soap” dimers formed through hydrogen bonding and “water-bridge” between the two species.^22^ It is the dimers that lead to the formation of bilayer structures, similar to the case of fatty acids in water.^27^ The relative contents of the three species change with *C* and pH. The pH of the KOCOOH solution decreases with the increase of *C* (Fig. S4, ESI†). With an increase in *C*, the contents of the three species all increase, but the ionization degree of KOCOOH (or the relative content of KOCOO^−^) decreases due to the decrease of pH. Within a much low *C* range (accompanied by relatively high pH), the dimers are the main specie, and their content first reaches the CVC of KOCOOH (corresponding to *C* ∼ 1.4 mM and pH ∼ 3.1) with the increase of *C*, resulting in the formation of vesicles.^22^ When *C* increases to a critical value (*ca.* 28 mM, pH ∼ 2.2), the total content of the dimer and monomeric KOCOOH species reaches their solubility limit while that of the monomeric KOCOO^−^ does not, due to the fact that the dimers and KOCOOH are more hydrophobic than KOCOO^−^ in nature. The excess dimers and KOCOOH form oil droplets, resulting in the microscopic phase separation (owing to the low *C*, no macroscopic phase separation appeared). With a further increase in *C* to another critical value (*ca.* 65 mM, pH ∼ 2.0), the total content of the two monomeric (KOCOOH and KOCOO^−^) species reaches the CMC of KOCOOH, leading to the formation of micelles, along with the disappearance of oil droplets. The micelles formed coexist with the vesicles in the system, resulting in the formation of ATPSs. Sakai *et al.*^28^ reported that monopotassium monododecyl phosphate (MAP-12K) in water form vesicles at a low *C*, which coexist with precipitates formed by dimers, but the vesicles are translated to micelles at a higher *C*, accompanied by the disappearance of precipitates. Our results are similar to the case of MAP-12K. Note that the decrease in pH caused by the increase of *C* is conducive to the formation of bilayer structures, because the low ionization degree of carboxylic acid groups can reduce the electrostatic repulsion between polar groups in aggregates. Therefore, the volume of the lower (vesicle) phase increases with the increase of *C* for the ATPS, due to the increased KOCOOH molecules being mainly participated in the formation of vesicles. Also, this is the reason of the transition from vesicles to strip bilayers and planar lamellar phase when *C* increases to 160 and 500 mM (corresponding to pH ∼ 1.7 and 1.4), respectively.

## 4. Conclusions

In conclusion, we report for the first time that ATPSs can form in the single-component aqueous solution of KOCOOH without any additives. The formation of the ATPSs arises from the coexistence of micelles and vesicles in the system. Their upper and lower phases consist of micelles and vesicles, respectively. In addition, a concentration-driven stepwise aggregation was identified for the KOCOOH solution. With an increase in its concentration, vesicles, oil droplets, micelles, strip bilayers, and planar lamellar phase form successively, which can be attributed to the constantly changing ionization state of KOCOOH in water; macroscopically, the system exhibit homogeneous transparent single-phase, turbid dispersion, two phases, bluish single-phase, and colorless transparent single-phase in turn. This work deepens the understanding of ATPSs, and the ATPS formed by KOCOOH may have potential applications such as in the separation and purification of biomolecules and the construction of hierarchical protocell models.

## Conflicts of interest

There are no conflicts to declare.

## Supplementary Material

RA-011-D1RA06474F-s001
