# Supplementary material for: An aqueous two-phase system formed in single-component solution of α-ketooctanoic acid
Source: RSC Adv. 2021 Oct 21;11(54):34245–9. doi: 10.1039/d1ra06474f (PMC9042353; doi:10.1039/d1ra06474f)
Supplement: RA-011-D1RA06474F-s001 [file RA-011-D1RA06474F-s001.pdf]

Electronic Supplementary Material (ESI) for RSC Advances.
This journal is © The Royal Society of Chemistry 2021

Electronic Supplementary Information

An aqueous two-phase system formed in single-component solution of α-ketooctanoic acid

Huifang Xu, [a] Xin Liang, [a] Yaping Zhang, [a] Meihua Gao, [b] Na Du, [b] Wanguo Hou*[b]

[a] College of Pharmacy, Henan University of Chinese Medicine, Zhengzhou 450046, P. R. China

[b] Key Laboratory of Colloid and Interface Chemistry (Ministry of Education), Shandong University, Jinan 250100, P. R. China

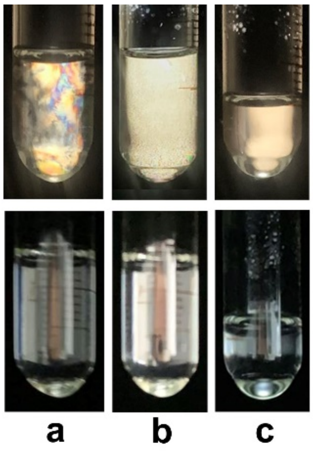

Fig. S1. Photographs of KOCOOH solutions at (a) 500, (b) 750, (c) 1000 mM, with (up) and without (below) crossed polarizers.

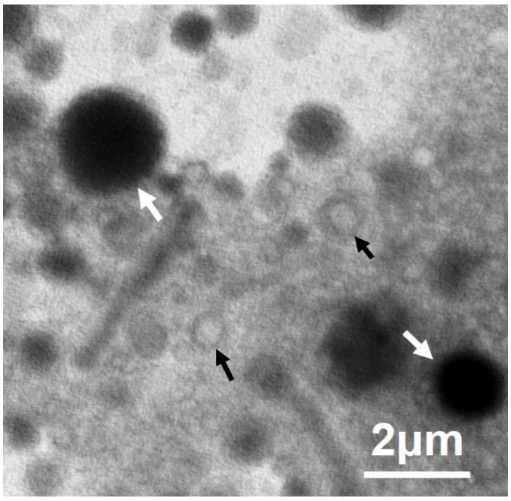

Fig. S2. NS-TEM image of KOCOOH solution with 50 mM. Vesicles and oil droplets, as indicated by black and white arrows, respectively, coexist in the system.

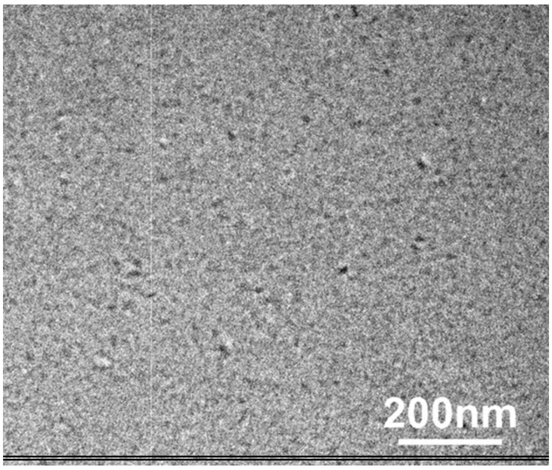

Fig. S3. Cryo-TEM image of the upper phase of ATPS with 130 mM.

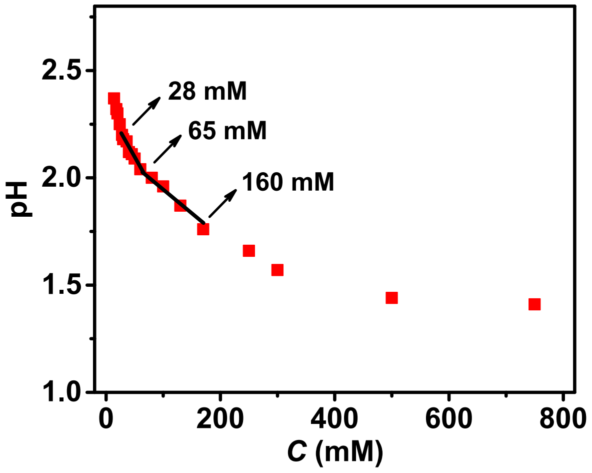

Fig. S4. Change of pH of KOCOOH solution with its concentration at 25 °C.
